# A systematic review of interventions to increase the use of smoking cessation services for women who smoke during pregnancy

**DOI:** 10.1111/ajo.13745

**Published:** 2023-08-25

**Authors:** Cheryl Bailey, Poliana de Barros Medeiros, David A. Ellwood, Philippa Middleton, Christine J. Andrews, Vicki J. Flenady

**Affiliations:** ^1^ Centre of Research Excellence in Stillbirth, Mater Research Institute The University of Queensland Brisbane Queensland Australia; ^2^ Department of Paediatrics and Neonatology Sunshine Coast University Hospital Sunshine Coast Queensland Australia; ^3^ School of Medicine & Dentistry Griffith University and Gold Coast University Hospital Gold Coast Queensland Australia; ^4^ South Australian Health and Medical Research Institute (SAHMRI) : Women and Kids and The University of Adelaide Adelaide South Australia Australia

**Keywords:** pregnancy, referral and consultation, smoking cessation, systematic review, tobacco

## Abstract

**Background:**

Although many pregnant women accept referrals to stop‐smoking support, the uptake of appointments often remains low.

**Aim:**

The aim was to review the success of interventions to increase the uptake of external stop‐smoking appointments following health professional referrals in pregnancy.

**Materials and Methods:**

Embase, PubMed, Cochrane Central Register of Controlled Trials, Scopus and CINAHL were searched in February 2023 for studies with interventions to increase the uptake rates of external stop‐smoking appointments among pregnant women who smoke. Eligible studies included randomised, controlled, cluster‐randomised, quasi‐randomised, before‐and‐after, interrupted time series, case–control and cohort studies. Cochrane tools assessing for bias and Preferred Reporting Items for Systematic Reviews and Meta‐Analyses (PRISMA) guidelines were followed.

**Results:**

Two before‐and‐after studies were included, including a combined total of 1996 women who smoked during pregnancy. Both studies had a serious risk of bias, and meta‐analysis was not possible due to heterogeneity. One study testing carbon monoxide monitors and opt‐out referrals showed increased uptake of external stop‐smoking appointments, health professional referrals and smoking cessation rates compared to self‐identified smoking status and opt‐in referrals. Results were limited in the second study, which used carbon monoxide monitors, urinary cotinine levels and self‐disclosed methods to identify the smoking status with opt‐out referrals. Only post‐intervention data were available on the uptake of appointments to external stop‐smoking services. The number of health professional referrals increased, but change in smoking cessation rates was less clear.

**Conclusions:**

There is insufficient evidence to inform practice regarding strategies to increase the uptake of external stop‐smoking appointments by women during pregnancy.

## BACKGROUND

Maternal smoking is one of the most important modifiable risk factors for reducing adverse pregnancy outcomes,^1^ including low birth weight, preterm birth and stillbirth.^2^ These adverse outcomes have a profound and long‐lasting impact on parents, families and care providers, with stillbirth rates showing little improvement globally over the past two decades.^3^ Women who stop smoking in early pregnancy (up to the start of the second trimester) can reduce their risk of stillbirth and other adverse birth outcomes to that of a non‐smoker.^4^, ^5^, ^6^ Although maternal smoking in Australia had shown a downward trend from 15% in 2009 to 9% in 2020,^7^ women from low socio‐economic backgrounds, young women^8^ and Aboriginal and Torres Strait Islander women have disproportionately higher rates of smoking in pregnancy.^9^

A Cochrane systematic review of interventions to support pregnant women to stop smoking showed that feedback (use of carbon monoxide monitors or measures of urine cotinine levels), incentives and psychosocial counselling appear to be effective in increasing smoking cessation rates.^6^, ^10^ Nicotine replacement therapy (NRT) may also be effective in improving the smoking cessation rate.^11^ This suggests that women will have a better chance of quitting when engaging with a smoking cessation program that provides these interventions. Health professionals can follow the 5A's pathway, which prompts them to Ask, Advise, Assess, Assist and Arrange smoking cessation support for women.^12^ This internationally recognised framework promotes smoking cessation by giving women smoking cessation advice and referring them to a locally appropriate stop‐smoking service.

Many pregnant women accept a referral to stop‐smoking support; however, the uptake of appointments (and thus smoking quit rates) often remains low.^13^ A study from the UK shows a gap in accepting referrals, with only 14% of referred smokers attending one or more appointments with stop‐smoking services.^14^ In 2019, the Stillbirth Centre of Research Excellence (Stillbirth CRE) introduced the Safer Baby Bundle (SBB), which implements strategies to prevent stillbirths and other adverse perinatal outcomes across Australia.^15^ The SBB recommends carbon monoxide monitoring and opt‐out referrals to appropriate local stop‐smoking services. Preliminary surveys completed by postnatal women before the implementation of the SBB showed that only 11% of referred smokers attended one or more appointments with stop‐smoking support (unpublished data).

Carbon monoxide monitors and opt‐out referrals may increase identification and referral rates; however, women who are not ready to stop smoking may agree to a referral yet not follow with an external appointment.^13^ Smoking cessation support during pregnancy is identified as a key stillbirth and preterm birth prevention strategy.^2^, ^15^, ^16^ Many families could avoid the tragedy of stillbirth by ensuring pregnant women receive high‐quality smoking cessation support structured to meet their needs.

This systematic review aims to determine the efficacy of interventions to increase the uptake of external stop‐smoking support appointments following a health professional referral for women who smoke during pregnancy.

## MATERIALS AND METHODS

The methodology for this review complies with the Preferred Reporting Items for Systematic Reviews and Meta‐Analyses (PRISMA) guidelines.^17^ The study protocol was registered through PROSPERO (CRD42021237279) and accepted on 17 March 2021.

### Types of studies

All relevant randomised controlled trials, cluster‐randomised trials, quasi‐randomised controlled trials, controlled before‐and‐after, interrupted time series, case–control and cohort studies were included.

### Population

Pregnant women accessing antenatal care at any stage in pregnancy have been identified as smokers. Smoking is defined as all products that contain nicotine – cigarettes, cigars, pipes, e‐cigarettes containing nicotine, tobacco that is chewed, sucked, dissolved or inhaled and snuff or snus.

### Intervention

Interventions to increase appointment uptake at external stop‐smoking support following health professional referral were reviewed in this study. External stop‐smoking support is defined as *a service not connected to or in the same organisation as the referring health professional*.

### Included interventions

• Supplementary health professional education on smoking cessation
• Opt‐out referral to smoking cessation support
• Opt‐out provision of stop‐smoking support
• Carbon monoxide monitoring
• Pre‐referral pharmacological support
• Pre‐referral behavioural support aimed at increasing appointment uptake with smoking cessation service

### Excluded interventions

• Financial incentives
• Smoking cessation support provided in the maternity setting by a health professional

Smoking cessation support provided face to face by a health professional.

### Comparator

Health professionals opt‐in referral to external smoking cessation support, for women identified as smoking while attending routine pregnancy care.

### Outcome measures

#### Primary outcome

• Change in the uptake rate of appointments with external stop‐smoking support following health professional referral, defined as attending one or more appointments after a referral

#### Secondary outcomes

• Change in the rate of health professional referrals to external smoking cessation support, defined as being referred to stop‐smoking support, as reported by the study investigators
• Change in the rates of smoking cessation, defined as smoking cessation during pregnancy and up to six weeks postpartum
• Women's experiences of interventions
• Women's experiences of stop‐smoking services

### Search strategy

Electronic databases Embase (Elsevier), MEDLINE (PubMed), Cochrane Central Register of Controlled Trials, Scopus (Elsevier) and CINAHL (EBSCO) were searched on 4 February 2023. Only studies published in English were included, and no publication dates were imposed.

Search terms were pilot tested on PubMed using MESH terms and subject headings and individualised for each database. The keywords included referrals OR referring OR referred OR uptake OR engag* ‘smoking cessation’ OR quitline OR ‘stop smoking’ AND Pregnan* OR ‘Prenatal care’ OR antenatal OR Gestation* OR Trimester* OR ‘Expectant mother*’ OR ‘Expecting mother*’ OR ‘Expectant wom*’ OR ‘Expecting wom*’. Full search terms are provided in Appendix S1.. Studies published in languages other than English, case reports, conference abstracts, letters, studies duplicating validation data (systematic reviews and meta‐analysis) from previous studies, grey literature and unpublished studies were excluded.

### Data collection and analysis

The electronic database search was performed using EndNote x9 and Covidence software.^18^ Initial search results were combined from the five databases, duplicates were removed and all the remaining articles were uploaded to Covidence. Two authors (author one and author two) independently screened all abstracts and/or titles. Full‐text articles were obtained for those studies where a decision could not be made based on the title and abstract. Two reviewers (author one and author two) undertook data extraction and quality assessment. Any uncertainties or conflicts were discussed and resolved with a third (author three) and fourth reviewer (author four).

### Quality assessment

The Cochrane Risk of Bias tools^19^, ^20^ were used by two reviewers (author one and author two) to assess the risk of bias. The risk of bias (RoB2) tool assessed the randomised studies, and the non‐randomised studies of interventions tool (ROBINS‐I) assessed the non‐randomised studies. Each domain was given an overall assessment of potential bias with scores of Low/Moderate/Serious/Critical/No Information. Studies were not excluded on the grounds of risk of bias.

## RESULTS

**Figure 1:**
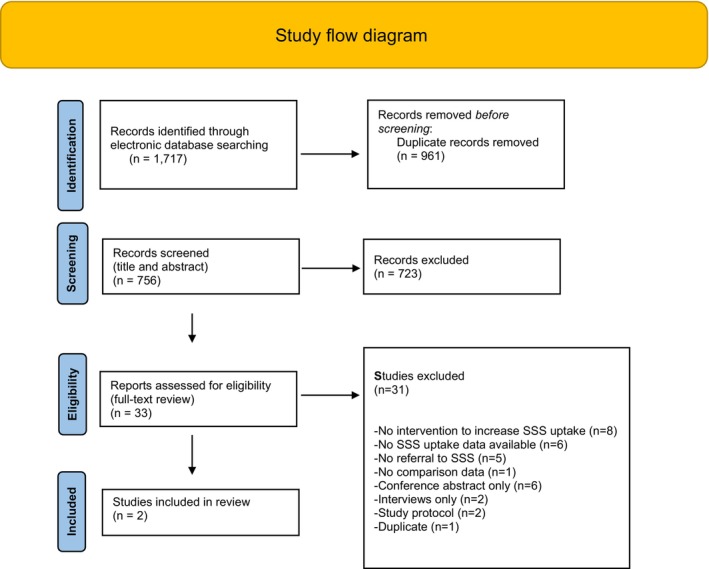
Search results, study selection and inclusion process. SSS, stop‐smoking service.

The initial search yielded 1717 potentially eligible studies. After duplicates were removed, 756 unique articles were transferred to Covidence and assessed against eligibility. Thirty‐three studies were reviewed in full text as potentially eligible for the review, with 31 studies excluded for the reasons described in Figure 1. Only two studies met the criteria for inclusion in the review. Meta‐analysis could not be performed due to the lack of data and heterogeneity in the interventions and the study sites.

### Characteristics of included studies

The review included two before‐and‐after studies,^13^, ^21^ both conducted in the UK, with a combined total of 1996 pregnant women identified as smoking. The study by Campbell *et al*.^21^ recruited 1216 participants in two sites, Kings Mill Hospital and Sherwood Women's Centre, in 2012/2013. The second study by Bauld *et al*.^13^ recruited 780 participants in a hospital in Dudley over eight months and a community maternity setting in South Birmingham over six months in 2010/2011. Campbell *et al*. collected data from the same periods (May to October) one year apart. Bauld *et al*. used the data reported for the year before the intervention to compare to post‐intervention. Campbell *et al*. recorded stop‐smoking service referrals, uptake and four‐week quit rates, while Bauld *et al*. recorded referrals and four‐week quit rates. Post‐intervention, the external stop‐smoking service uptake rates were recorded; however, no pre‐implementation data on uptake rates were documented. No data were available on the secondary outcomes and women's experiences of interventions and attending smoking cessation support.

### Participant characteristics

Across both studies, 6005 pregnant women accessed maternity care post‐intervention, 2293 in the study by Campbell *et al*. and 3712 in the study by Bauld *et al*. Overall, 24% (*n* = 1460) of women were identified as smoking while pregnant, with Campbell *et al*. identifying 30% (*n* = 680) and Bauld *et al*. 22% (*n* = 780) as smokers. Both studies were implemented in areas of socio‐economic deprivation with a high smoking population (see Table 1). Participants’ ethnicity and maternal age were not recorded in either study.

**Table 1:** Characteristics of included studies

Author	Country and year	Smoking prevalence	Socio‐economic status	Study type	Intervention	Comparator	Woman's experience of intervention	Women's experience of stop‐smoking service
Bauld *et al*.^13^	UK 2010–2011	High	Low	Before and after	Carbon monoxide test/urinary cotinine/self‐disclosed smoking status and opt‐out referral at the eight‐week midwife appointment and the 12‐week ultrasound scan	Self‐disclosed smoking status and opt‐in referral	Not reported	Not reported
Campbell *et al*.^21^	UK 2012–2013	High	Low	Before and after	Carbon monoxide test and opt‐out referral at the 12‐week ultrasound scan	Self‐disclosed smoking status and opt‐in referral	Not reported	Not reported

### Interventions

Campbell *et al*. used carbon monoxide monitors to identify smokers and opt‐out referrals to external stop‐smoking services at the routine 12‐week ultrasound scan and found that carbon monoxide monitoring identified more pregnant women who smoke when compared to women self‐reporting smoking status. Pre‐intervention, 23% (*n* = 536) of women self‐reported smoking at the booking appointment compared to 30% (*n* = 680) post‐intervention. Bauld *et al*. identified smokers using several approaches, including self‐reported, carbon monoxide and urinary cotinine methods, followed by an opt‐out referral to external stop‐smoking services. This was implemented at the eight‐week midwife appointment or the 12‐week ultrasound scan.

### Risk of bias

Both studies showed a serious risk of bias in one or more areas (see Table 2). The study by Campbell *et al*. showed a serious risk of bias in the measurement of outcomes. Pre‐intervention referrals were completed in routine antenatal care, and post‐intervention referrals were completed at the 12‐week ultrasound scan after the woman had seen an image of her baby. This change could increase the proportion of women attending external stop‐smoking service appointments and was not accounted for in the study. All other areas of bias were assessed as moderate. This study was not randomised, and there were no identified missing data; most smokers were enrolled into the study using carbon monoxide monitors, and data comparison aligned with the same period in the previous year. Bias was assessed to be serious in one area and moderate in the other six, resulting in a judgement of serious risk of bias overall.

**Table 2:** Risk of bias assessment

	Bias due to confounding	Selection of participants into the study	Bias in classification of interventions	Bias due to deviations from intended interventions	Bias due to missing data	Bias in measurement of outcomes	Bias in selection of the reported result
Bauld *et al*.	Moderate	Moderate	Serious	Moderate	Serious	Serious	Moderate
Campbell *et al*.	Moderate	Moderate	Moderate	Moderate	Moderate	Serious	Moderate

The study by Bauld *et al*. showed a serious risk of bias in three areas and thus a serious risk of bias overall. The areas were as follows:

• Classification of intervention (as not all eligible women were referred).
• Missing data, due to the lack of quality in pre‐intervention data.
• Outcomes measurement, with some women not receiving a referral from the health professional, as they were already in touch with stop‐smoking support but were included in the uptake numbers in the study.

A summary of the bias assessment is presented in Table 2.

### Primary outcome

#### Change in the uptake of external stop‐smoking support appointments

Campbell *et al*. reported that 2.5% (95% confidence interval (CI): 1.9–3.2%, *P* < 0.001, *n* = 57) of pregnant women attended one or more appointments with stop‐smoking support pre‐intervention, compared to 5.3% (95% CI: 4.4–6.3%, *P* < 0.001, *n* = 121) post‐intervention, showing an increase of 112%. Bauld *et al*. reported that 3.5% of pregnant women (*n* = 129) attended one or more appointments with stop‐smoking support post‐intervention (see Table 3). Due to the lack of pre‐implementation data, it was not possible to report uptake rates.

**Table 3:** Results showing referral rates and uptake of external stop‐smoking support

	Study timepoint	Intervention	Pregnant women eligible to participate in the study, *n*	Identified smokers, *n* (%)	Declined referral, *n* (%)	Referral, *n* (%)	Already in touch with stop‐smoking services, *n* (%)	Uptake of stop‐smoking services, *n* (%)	Four‐week cessation rate of pregnant women eligible to participate in the study, *n* (%)
Bauld *et al*.'s^13^ pilot study from October 2010 to March 2011	Routine data collected before the implementation of the pilot study from October 2009 to March 2010	Routine antenatal care (self‐identified smoking status and opt‐in referral)	Not reported	Not reported	Not reported	687	Not reported	Not reported	97
	Routine data collected during the implementation of the pilot study from October 2010 to March 2011	Opt‐out referral of identified smokers	Not reported	Not reported	Not reported	833	Not reported	Not reported	86
	Data were collected from maternity bookings and the stop‐smoking service during a pilot study from October 2010 to March 2011	Opt‐out referral of identified smokers	3712	780 (21%)	312 (8.4%)	291 (7.8%)	159 (4.3%)	129 (3.5%)^†^	51 (1.4%)^†^
Campbell *et al*.'s^21^ service evaluation from May 2012 to October 2013	Data were collected between May and October 2012, prior to the intervention	Routine antenatal care (self‐identified smoking status and opt‐in referral)	2287	536 (23.4%)	Not reported	209 (12.7%)	Not reported	57 (2.5%)	46 (2%)
	Data were collected between May and October 2013, during the intervention	Carbon monoxide monitoring and opt‐out referrals	2293	680 (29.7%)	Not reported	421 (18.4%)	Not reported	121 (5.3%)	93 (4.1%)

† Includes women already in touch with the stop‐smoking service.

### Secondary outcomes

#### Change in the uptake of opt‐out referrals to external stop‐smoking support

Campbell *et al*. reported that opt‐out referrals to external stop‐smoking support increased the rate of referrals compared to pre‐intervention rates. Pre‐intervention, 12.7% (95% CI: 11.4–14.1%, *P* < 0.001, *n* = 209) of pregnant women were referred to stop‐smoking support compared to 18.4% (95% CI: 16.8–20.0%, *P* < 0.001, *n* = 421) post‐intervention, showing an increase of 45%. Bauld *et al*. used routinely collected data to compare pre‐ and post‐intervention referral rates over the same six‐month period from October to March 2009 to 2011. More women were referred to stop‐smoking services post‐intervention compared to the service's quarterly data from the previous year (see Table 3).

#### Change in smoking cessation rates

Campbell *et al*. reported 2% (95% CI: 1.5–2.7%, *P* < 0.001, *n* = 46) of pregnant women eligible to participate in the study had stopped smoking four weeks after contact with the stop‐smoking service compared to 4.1% (95% CI: 3.3% to 4.9%, *P* < 0.001, *n* = 93) post‐intervention, showing an increase of 105%. Bauld *et al*. reported that 1.4% (*n* = 51) of pregnant women eligible to participate in the study had stopped smoking four weeks after contact with the stop‐smoking service post‐intervention. Unfortunately, there were no pre‐implementation data recorded for comparison. Bauld *et al*. also used routinely collected data to compare the four‐week pre‐ and post‐intervention quit rates over the same six‐month period from October to March; however, the number of pregnant women who were eligible for referral was not reported. In 2009/2010 pre‐intervention, *n* = 97 of the women who were referred had stopped smoking four weeks after contact with the stop‐smoking services compared to *n* = 86 of the women who were referred in 2010/2011 post‐intervention (see Table 3).

No data were available on the secondary outcomes and women's experiences of interventions and of attending smoking cessation support.

## DISCUSSION

To the best of our knowledge, this is the first systematic review to investigate the efficacy of interventions to increase the uptake of external stop‐smoking support appointments following a health professional referral of women who smoke during pregnancy. However, small participant numbers, heterogeneity of outcome measures and low study quality limit review findings.

A combined total of 1996 pregnant women who smoke were included in this review. The results from Campbell *et al*. are encouraging, indicating that carbon monoxide monitoring may benefit the identification of smokers and that opt‐out referrals increase the number of women referred to external stop‐smoking support. However, whether this intervention increased external stop‐smoking support appointment uptake and smoking cessation rates is unclear. In addition, Bauld *et al*. reported that some women were already in contact with external stop‐smoking support services and did not receive a referral but were included in appointment uptake numbers. The study did not account for this; therefore, the appointment uptake of women referred by a health professional and smoking cessation rates could be much lower. Unfortunately, no data were reported on women's experiences of the intervention or attendance at external stop‐smoking support, so no recommendations could be made.

Both studies show that only a small proportion of pregnant women who smoke were referred to external stop‐smoking support services, and even fewer attended their appointment with external stop‐smoking services. These low rates of stop‐smoking support uptake following referral have also been reported in the 2018 evaluation of the Saving Babies Lives Care Bundle from the UK^22^ and the pre‐implementation data from the SBB in Australia (unpublished data). This may be due to referrals accepted by women who are not ready to make the change.^13^ A lack of routinely collected data on referral and uptake of smoking cessation services globally and in Australia prevents robust analysis. Further effective monitoring of service delivery and process outcomes are needed to inform the development of strategies to increase smoking cessation support uptake.

Both of the included studies found that more pregnant women who smoke could be identified when carbon monoxide monitors and urinary cotinine tests were implemented to determine their smoking status. An increase in the identification of smokers using carbon monoxide monitors in pregnancy has also been reported in other studies.^21^, ^23^ Carbon monoxide monitors can remove the fear of disclosing, with some studies showing that carbon monoxide testing is acceptable for many women as part of their routine pregnancy care.^24^, ^25^ Health professionals may also find testing helpful when educating women about smoking during pregnancy, as this can eliminate the awkward conversations asking women about their smoking status,^26^ and visual feedback on carbon monoxide levels can provide a powerful motivator for quitting.^27^ However, some midwives reported feeling concerned that testing may affect their relationship with women,^27^, ^28^ as screening such as this makes some women think they cannot be trusted to disclose that they smoke.^29^

The opt‐out approach increases the number of referrals in both studies compared to the opt‐in referrals. Bauld *et al*. reported an increase in referrals compared to the previous year, with Campbell *et al*. reporting double the number of women referred. This increase in referral rates has also been described in other studies.^23^, ^30^ Implementing the opt‐out referrals at the 12‐week ultrasound scan may have contributed to the increased referral rates in both studies included in this review. However, the motivating factor of seeing the baby on the scan was not accounted for in either analysis. Therefore, we could not identify whether the ultrasound scan was the motivating factor associated with increased referral and uptake rates, as seen in a 2009 US study by Stotts *et al*.,^31^ which found that smoking cessation increased in light smokers when motivational interviewing was provided at a second‐ or third‐trimester ultrasound scan.

Socio‐economic factors may have influenced the uptake of referral and quitting in the included studies. In several countries, including USA,^32^ Britain, Finland,^33^ China, Ghana, India and South Africa,^34^ lower socio‐economic factors have been linked to greater use of tobacco in members of the general public. Low socio‐economic status has also been associated with increased tobacco use and the reduced likelihood of quitting smoking in pregnancy.^35^ This could have reduced the potential number of women who stopped smoking from the studies in this review. Interventions that are personalised and sensitive to the needs of women who face barriers posed by complex socio‐economic disadvantages may improve outcomes.^36^ Financial incentives offered to low‐income pregnant women have shown improvements in engagement and treatment,^37^ and The Breathe Study showed favourable results with a clinic‐based specialist service, offering a face‐to‐face appointment with a specialist smoking cessation midwife.^38^ However, further research is needed on strategies to help women uptake external appointments with stop‐smoking services.

In 2018, a systematic review published in *The Lancet* estimated the global prevalence of smoking during pregnancy to be around 2%, varying between 8.1% (95% CI: 4.0–12.2) in the European Region and 0.8% (0.0–2.2) in the African Region. Approximately 53% (95% CI: 45.6–60.3) of these women continue to smoke daily during pregnancy.^39^ In 2020, 9% of women reported smoking while pregnant in Australia, with a higher rate of 43% observed among Indigenous mothers.^7^ A 2016 systematic review estimated that around 87% of pregnant women who smoked tried to quit and were unsuccessful, or did not attempt to stop,^40^ showing the need for improvements in current cessation supports. Offering referrals to external stop‐smoking services at all antenatal appointments could improve the rate of appointment uptake further. Campbell *et al*.^30^ identified that a significant number of women who initially opted out of the referral would have liked to receive further stop‐smoking service referrals at subsequent antenatal appointments.

### Strengths and limitations

The strengths of this review include the rigorous, pre‐defined methods and a comprehensive search strategy to include all relevant studies. However, the small number and the low quality of the studies included in the review allow only limited conclusions on interventions to increase the uptake of external stop‐smoking support.

Both studies found that most maternity services could integrate opt‐out referral pathways into care. However, both studies were performed in areas with higher‐than‐average smoking rates in disadvantaged neighbourhoods. Therefore, these results may only be generalisable in regions that provide carbon monoxide monitoring and opt‐out referrals at appointments offering ultrasound scans.

## CONCLUSION

Due to the limited number of studies available on interventions to increase external stop‐smoking support appointment uptake, there needs to be more evidence to inform future practice. There is some indication that carbon monoxide monitoring may benefit the identification of smokers; however, evidence on interventions that increase external stop‐smoking support referrals, external stop‐smoking support uptake and smoking cessation rates is less clear. In addition, no data were reported on women's experiences of the intervention and external stop‐smoking services. Further high‐quality research is needed on what will prepare women to make the change to enable the uptake of appointments with external stop‐smoking services after referral. Research on how health professionals can support women to prepare to make the change is also needed.

## FUNDING

This work was supported by The NHMRC Stillbirth Centre of Research Excellence and The University of Queensland Australian Government Research Training Program Scholarship.

## ETHICAL STATEMENT

Ethics approval was not needed for this literature review.

## AUTHOR CONTRIBUTIONS

C.B. and P.B.M. independently screened all abstracts and/or titles and full‐text articles. C.B. and P.B.M. undertook data extraction and quality assessment. C.B. performed data analysis and prepared the manuscript. C.A., P.B.M., D.E., P.M. and V.F. assisted in the conception and design of the study and reviewed the manuscript. All authors have approved the final version of the manuscript.

## Supporting information


**Appendix S1.** Search strategy.
