# Supplementary material for: A systematic review of interventions to increase the use of smoking cessation services for women who smoke during pregnancy
Source: Aust N Z J Obstet Gynaecol. 2023 Aug 25;63(6):737–45. doi: 10.1111/ajo.13745 (PMC10952730; doi:10.1111/ajo.13745)
Supplement: Supplementary file 1 — Appendix S1. Search strategy. [file AJO-63-737-s001.docx]

**Appendix S1**

**Search strategy**

**PubMed**

(referral[tiab] OR referrals[tiab] OR referring[tiab] OR referred[tiab] OR "Referral and Consultation"[Mesh] OR uptake[tiab] OR engag*[tiab] OR utilis*[tiab] OR utiliz*[tiab] OR "Patient Acceptance of Health Care"[Mesh])
AND
("smoking cessation"[tiab] OR "tobacco cessation"[tiab] OR quitline[tiab] OR "stop smoking"[tiab] OR "quit smoking"[tiab] OR "smoking reduction"[tiab] OR "Smoking Cessation"[Mesh] OR "Tobacco Use Cessation"[Mesh] OR "Smoking Reduction"[Mesh])
AND
(Pregnan*[tiab] OR “Prenatal care”[tiab] OR Gestation*[tiab] OR Trimester*[tiab] OR “Expectant mother*” [tiab] OR “Expecting mother*”[tiab] OR “Expectant wom*”[tiab] OR “Expecting wom*”[tiab] OR Pregnancy[MeSH] OR "Pregnancy Trimesters"[MeSH] OR antenatal[tiab] OR "Pregnant Women"[MeSH] OR "Prenatal care"[MeSH])

**Embase - DH translation**

(referral:ti,ab OR referrals:ti,ab OR referring:ti,ab OR referred:ti,ab OR 'patient referral'/exp OR uptake:ti,ab OR engag*:ti,ab OR utilis*:ti,ab OR utiliz*:ti,ab OR 'patient participation'/exp)

AND

("smoking cessation":ti,ab OR "tobacco cessation":ti,ab OR quitline:ti,ab OR "stop smoking":ti,ab OR "quit smoking":ti,ab OR "smoking reduction":ti,ab OR 'smoking cessation'/exp OR 'smoking reduction'/exp)

AND

(Pregnan*:ti,ab OR "Prenatal care":ti,ab OR Gestation*:ti,ab OR Trimester*:ti,ab OR "Expectant mother*":ti,ab OR "Expecting mother*":ti,ab OR "Expectant wom*":ti,ab OR "Expecting wom*":ti,ab OR 'pregnancy'/exp OR antenatal:ti,ab OR 'pregnant woman'/exp OR 'prenatal care'/exp)

**CENTRAL** **DH translation**(referral:ti,ab OR referrals:ti,ab OR referring:ti,ab OR referred:ti,ab OR [mh "Referral and Consultation"] OR uptake:ti,ab OR engag*:ti,ab OR utilis*:ti,ab OR utiliz*:ti,ab OR [mh "Patient Acceptance of Health Care"])

AND

("smoking cessation":ti,ab OR "tobacco cessation":ti,ab OR quitline:ti,ab OR "stop smoking":ti,ab OR "quit smoking":ti,ab OR "smoking reduction":ti,ab OR [mh "Smoking Cessation"] OR [mh "Tobacco Use Cessation"] OR [mh "Smoking Reduction"])

AND

(Pregnan*:ti,ab OR "Prenatal care":ti,ab OR Gestation*:ti,ab OR Trimester*:ti,ab OR (Expectant NEXT mother*):ti,ab OR (Expecting NEXT mother*):ti,ab OR (Expectant NEXT wom*):ti,ab OR (Expecting NEXT wom*):ti,ab OR [mh Pregnancy] OR [mh "Pregnancy Trimesters"] OR antenatal:ti,ab OR [mh "Pregnant Women"] OR [mh "Prenatal care"])

**CINAHL** - DH translation
((TI referral OR AB referral) OR (TI referrals OR AB referrals) OR (TI referring OR AB referring) OR (TI referred OR AB referred) OR (MH "Referral and Consultation+") OR (TI uptake OR AB uptake) OR (TI engag* OR AB engag*) OR (TI utilis* OR AB utilis*) OR (TI utiliz* OR AB utiliz*))

AND

((TI "smoking cessation" OR AB "smoking cessation") OR (TI "tobacco cessation" OR AB "tobacco cessation") OR (TI quitline OR AB quitline) OR (TI "stop smoking" OR AB "stop smoking") OR (TI "quit smoking" OR AB "quit smoking") OR (TI "smoking reduction" OR AB "smoking reduction") OR (MH "Smoking Cessation+") OR (MH "Smoking Cessation Programs+"))

AND

((TI Pregnan* OR AB Pregnan*) OR (TI "Prenatal care" OR AB "Prenatal care") OR (TI Gestation* OR AB Gestation*) OR (TI Trimester* OR AB Trimester*) OR (TI "Expectant mother*" OR AB "Expectant mother*") OR (TI "Expecting mother*" OR AB "Expecting mother*") OR (TI "Expectant wom*" OR AB "Expectant wom*") OR (TI "Expecting wom*" OR AB "Expecting wom*") OR (MH "Pregnancy+") OR (MH "Pregnancy Trimesters+") OR antenatal OR (MH "Expectant Mothers+") OR (MH "Prenatal care+"))

**Scopus,**

(referrals OR referring OR referred OR uptake OR engag*)
AND
("smoking cessation" OR quitline OR "stop smoking")
AND
(Pregnan* OR “Prenatal care” OR Gestation* OR Trimester* OR “Expectant mother*” OR “Expecting mother*” OR antenatal OR “Expectant wom*” OR “Expecting wom*”)

**Scopus - DH translation**
(TITLE-ABS("referral") OR TITLE-ABS("referrals") OR TITLE-ABS("referring") OR TITLE-ABS("referred") OR INDEXTERMS("Referral and Consultation") OR TITLE-ABS("uptake") OR TITLE-ABS("engag*") OR TITLE-ABS("utilis*") OR TITLE-ABS("utiliz*") OR INDEXTERMS("Patient Acceptance of Health Care"))

AND

(TITLE-ABS("smoking cessation") OR TITLE-ABS("tobacco cessation") OR TITLE-ABS("quitline") OR TITLE-ABS("stop smoking") OR TITLE-ABS("quit smoking") OR TITLE-ABS("smoking reduction") OR INDEXTERMS("Smoking Cessation") OR INDEXTERMS("Tobacco Use Cessation") OR INDEXTERMS("Smoking Reduction"))

AND

(TITLE-ABS("Pregnan*") OR TITLE-ABS("Prenatal care") OR TITLE-ABS("Gestation*") OR TITLE-ABS("Trimester*") OR TITLE-ABS("Expectant mother*") OR TITLE-ABS("Expecting mother*") OR TITLE-ABS("Expectant wom*") OR TITLE-ABS("Expecting wom*") OR INDEXTERMS("Pregnancy") OR INDEXTERMS("Pregnancy Trimesters") OR "antenatal" OR INDEXTERMS("Pregnant Women") OR INDEXTERMS("Prenatal care"))
